# MicroRNA-214 enriched exosomes from human cerebral endothelial cells (hCEC) sensitize hepatocellular carcinoma to anti-cancer drugs

**DOI:** 10.18632/oncotarget.27879

**Published:** 2021-02-02

**Authors:** Louie Semaan, Qingning Zeng, Yong Lu, Yi Zhang, Mehdi Mohamad Zreik, Mohamad Baqer Chamseddine, Michael Chopp, Zheng Gang Zhang, Dilip Moonka

**Affiliations:** ^1^Department of Neurology, Henry Ford Hospital, Detroit, MI 48202, USA; ^2^Division of Gastroenterology and Hepatology, Henry Ford Hospital, Detroit, MI 48202, USA; ^3^Department of Physics, Oakland University, Rochester, MI 48309, USA; ^4^Department of Neurology, School of Medicine, Wayne State University, Detroit, MI 48202, USA; ^5^Department of Natural Sciences, University of Michigan–Dearborn, Dearborn, MI 48128, USA; ^*^These authors contributed equally to this work; ^#^Contributed equally as senior authors

**Keywords:** hepatocellular carcinoma (HCC), human cerebral endothelial cells-derived exosomes, microRNA-214, therapeutic efficacy, anti-cancer drug

## Abstract

Hepatocellular carcinoma (HCC) is the most common primary liver tumor worldwide. Current medical therapy for HCC has limited efficacy. The present study tests the hypothesis that human cerebral endothelial cell-derived exosomes carrying elevated miR-214 (hCEC-Exo-214) can amplify the efficacy of anti-cancer drugs on HCC cells. Treatment of HepG2 and Hep3B cells with hCEC-Exo-214 in combination with anti-cancer agents, oxaliplatin or sorafenib, significantly reduced cancer cell viability and invasion compared with monotherapy with either drug. Additionally, the therapeutic effect of the combination therapy was detected in primary tumor cells derived from patients with HCC. The ability of hCEC-Exo-214 in sensitizing HCC cells to anti-cancer drugs was specific, in that combination therapy did not affect the viability and invasion of human liver epithelial cells and non-cancer primary cells. Furthermore, compared to monotherapy with oxaliplatin and sorafenib, hCEC-Exo-214 in combination with either drug substantially reduced protein levels of P-glycoprotein (P-gp) and splicing factor 3B subunit 3 (SF3B3) in HCC cells. P-gp and SF3B3 are among miR-214 target genes and are known to mediate drug resistance and cancer cell proliferation, respectively. In conclusion, the present *in vitro* study provides evidence that hCEC-Exo-214 significantly enhances the anti-tumor efficacy of oxaliplatin and sorafenib on HCC cells.

## INTRODUCTION

Liver cancer is the fifth leading cause of cancer death worldwide. Hepatocellular carcinoma (HCC) is the dominant form of primary liver cancer, accounting for 85–90% of cases [1]. Surgical resection and liver transplantation are curative options but are offered to a small percentage of eligible patients [2]. Patients with advanced liver cancer, have limited medical options including sorafenib, which increases overall survival by less than three months [3]. Drug resistance is often evident within several months of initial treatment [4]. The low efficacy and high adverse side effect profile of currently available therapy provide a compelling need for more effective treatments [5].

MicroRNAs (miRNAs) are non-coding, small regulatory RNAs that target the 3′-untranslated region (3′-UTR) of mRNAs and negatively regulate gene expression at the post-transcriptional level [6, 7]. miRNAs are key players in cancer biology, affecting molecular pathways involved in cell proliferation, angiogenesis, invasion, metastasis, and apoptosis in cancer progression [8, 9]. miRNAs help mediate HCC pathogenesis [10, 11]. miR-214, specifically, appears to function as a tumor suppressor in HCC by inhibiting cell proliferation, invasion, angiogenesis, and metastasis [12, 13]. Down-regulation of miR-214 has been detected in patients with HCC and in HCC cell lines [14–16]. miR-214 targets genes including hepatoma-derived growth factor (HDGF) [17], fibroblast growth factor receptor 1 (FGFR1) [18], β-catenin [14], and Wnt3a [19]. Upregulation of miR-214, therefore, may have a potential therapeutic role in HCC. However, to date, systemic delivery of RNAs, including miRNAs, have had limited therapeutic efficacy in cancer therapy [20].

Exosomes are cell-derived extracellular nano-vesicles (~30–150 nm) found in a wide range of biofluids and tissues [21]. They transport biologically active molecules including proteins, lipids, DNA, and RNA as cargo and facilitate local and long-distance intercellular communication [22–25]. Although essentially all eukaryotic cell types produce exosomes, emerging evidence has shown that exosome cargos differ greatly depending on their cell of origin [26]. Tumor cell-derived exosomes are actively involved in metastatic niche formation, cancer progression, immune function regulation, and drug resistance [27–31]. Blood-derived exosomes are potential diagnostic and prognostic biomarkers for HCC [32–34]. In addition, use of exosomes as a vehicle to carry chemo-drugs, proteins, and miRNAs to treat cancer is being actively investigated [35–38]. The majority of intravenously administered exogenous exosomes are taken up by liver [39], making the use of exosomes to treat liver cancer especially appealing. Indeed, several studies have investigated the effect of engineered exosomes on liver cancer [40–42]. For example, mesenchymal stem cell (MSC)-derived exosomes carrying elevated miR-122 [43] and miR-199 [44] increase anti-cancer drug sensitivity of HCC cells to sorafenib and doxorubicin, respectively.

We previously demonstrated that engineered human cerebral endothelial cell-derived exosomes carrying elevated miR-214 (hCEC-Exo-214) sensitize ovarian cancer cells to chemotherapeutic agents [45]. In the present study, we test the hypothesis that miR-214 enriched exosomes can enhance the effect of anti-cancer drugs, oxaliplatin and sorafenib, on HCC cells.

## RESULTS

### Downregulation of miR-214 in hepatocellular carcinoma cells

**Figure 1:**
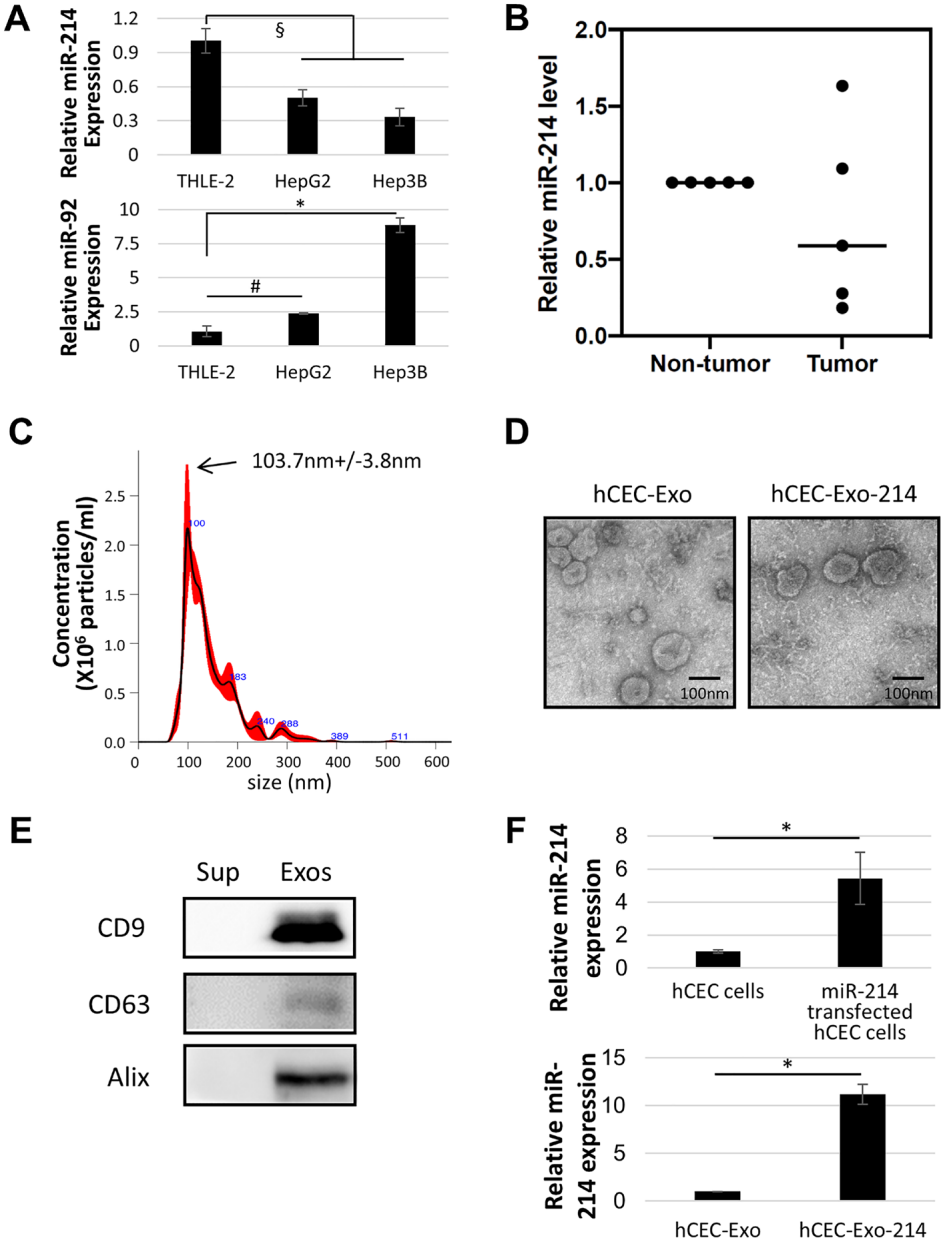
Characterization of miR-214 enriched exosomes. (**A**) Quantitative RT-PCR analysis of miR-214 and miR-92 levels in cell lines. (**B**) Quantitative RT-PCR analysis of miR-214 levels in tumor and non-tumor tissue of five individual HCC patients. (**C**) NTA analysis shows particle distributions of EVs isolated from culture medium of hCEC cells transfected by miR-214. (**D**) Representative TEM images of exosomes isolated from naïve (hCEC-Exo) and miR-214 transfected (hCEC-Exo-214) hCEC cells. (**E**) Representative Western blot images show exosomal marker proteins in supernatant (Sup) and exosomes (Exos). (**F**) Quantitative RT-PCR analysis shows miR-214 levels in non-transfected and transfected hCECs, and in exosomes isolated from naïve (hCEC-Exo) and miR-214 transfected (hCEC-Exo-214) hCEC cells. All mRNAs were normalized with U6 snRNA. Data are presented as mean ± SEM. *N* = 3. ^#^ *P* < 0.05, ^§^ *P* < 0.01, ^*^ *P* < 0.001.

miRNAs including miR-214 are involved in the development of HCC. We examined miR-214 expression in two HCC cell lines, HepG2 and Hep3B. Quantitative RT-PCR analysis showed that, compared to normal human liver epithelial cells (THLE-2), HepG2 and Hep3B cells exhibited an approximate 50 and 70% significant reduction in miR-214 expression, respectively, whereas miR-92 expression was upregulated in HCC cells (Figure 1A). Additionally, we measured miR-214 levels in liver tumor tissues acquired from patients with HCC who had undergone tumor resection. We found that, compared to non-tumor liver tissue from the same individuals, 3 of 5 (60%) tumor tissues showed reduced miR-214 levels (Figure 1B). These data are consistent with reports by others [14–16, 19, 46] and suggest that miR-214 is involved in HCC.

### Engineered hCEC-exosomes carrying elevated miR-214 (hCEC-Exo-214) enhance HCC sensitivity to anti-cancer drugs

Overexpression of miR-214 in SK-Hep1 cells inhibits tumor cell growth [14, 17]. Using hCEC-Exo-214, we have demonstrated that the engineered hCEC-Exo-214 sensitize ovarian cancer cells to chemotherapeutic agents [45]. hCEC-Exo-214 were isolated from the supernatant of hCECs transfected with a lentivector expressing pre-miR-214 by means of differential ultracentrifugation. Isolated extracellular vesicles had a mean size of 104 nm and exhibited donut-shaped morphology demonstrated by Nanoparticle Tracking Analysis (NTA) and transmission electron microscopy (TEM), respectively (Figure 1C and 1D). Western blot analysis revealed that these extracellular vesicles expressed exosomal marker proteins, CD9, CD63, and Alix (Figure 1E). Quantitative RT-PCR showed that, compared to non-transfected hCECs, hCECs transfected with pre-miR-214 had upregulated miR-214. hCEC-Exo-214 had an approximately 11-fold increase in miR-214 compared to naïve hCEC-Exo (Figure 1F).

**Figure 2:**
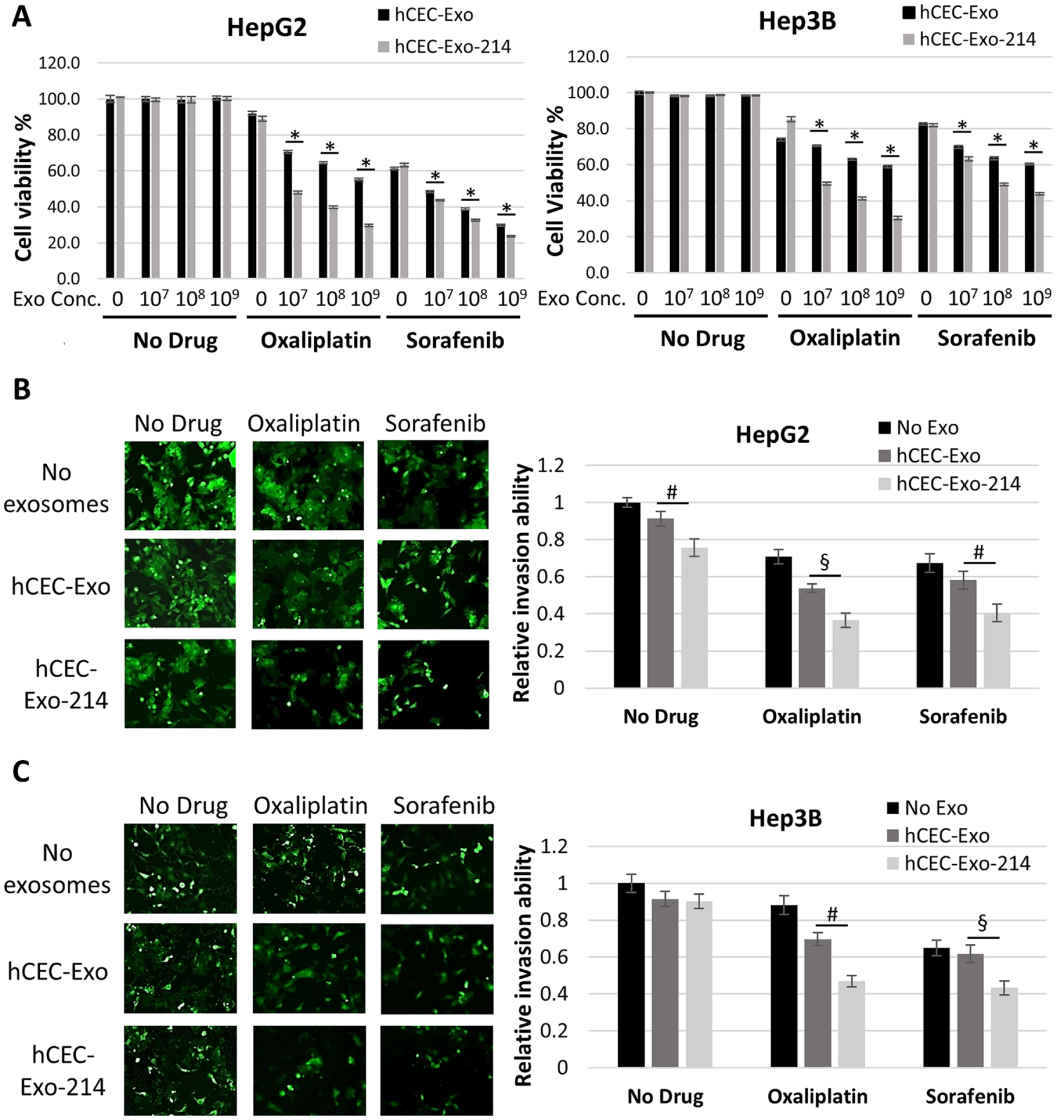
hCEC-Exo-214 sensitize HCC cells to anti-cancer drugs. (**A**) Cell viability of HepG2 and Hep3B cells treated with anti-cancer drugs and exosomes. Data are representative of three independent experiments. Values are expressed as mean ± SD. *N* = 5. (**B** and **C**) Representative images and quantitative data show cell invasion of HepG2 (B) and Hep3B (C) cells treated with hCEC-Exo or hCEC-Exo-214 in combination with oxaliplatin or sorafenib. Data are presented as mean ± SEM. *N* = 3. ^#^ *P* < 0.05, ^§^ *P* < 0.01, ^*^ *P* < 0.001.

hCEC-Exo-214, alone and in combination with anti-cancer drugs, were evaluated for their effect on HepG2 and Hep3B cells. Neither naïve hCEC-Exo nor hCEC-Exo-214 alone at doses of 10^7^, 10^8^, and 10^9^ particles/ml affected HCC cell viability measured by the MTT assay (Figure 2A). Oxaliplatin and sorafenib by themselves decreased cell viability of both HepG2 and Hep3B cells in a dose-dependent manner (Figure 2A and Supplementary Figure 1), consistent with previous reports [47–49]. Based on the dose response data, a dose at 0.0625 μM of oxaliplatin was selected for HepG2 and Hep3B cells, while a dose of 1.2 μM or 0.8 μM of sorafenib was selected for HepG2 or Hep3B cells, respectively (Supplementary Figure 1). Naïve hCEC-Exo and hCEC-Exo-214 were evaluated to determine whether they enhanced the effect of oxaliplatin or sorafenib on HCC viability. The MTT analysis showed that naïve hCEC-Exo or hCEC-Exo-214 in combination with oxaliplatin or sorafenib significantly reduced viable HepG2 and Hep3B cells in an exosomal concentration dependent manner with the most robust reduction at the highest concentration (10^9^ particles/ml) tested (Figure 2A). Importantly, compared to naïve hCEC-Exo, hCEC-Exo-214 further significantly reduced cancer cell viability (Figure 2A). In contrast, naïve hCEC-Exo or hCEC-Exo-214, in combination with oxaliplatin and sorafenib, did not significantly affect normal liver epithelial cell viability (Supplementary Figure 2), suggesting that the enhanced anti-cancer drug activity of combination treatment is specific to HCC tumor cells.

Next, the effect of combination therapy on HCC cell invasion was evaluated by means of a transwell cell invasion assay [50, 51]. The transwell assay analysis showed that neither naïve hCEC-Exo nor hCEC-Exo-214 alone significantly reduced cell invasion. However, naïve hCEC-Exo or hCEC-Exo-214, in combination with oxaliplatin or sorafenib significantly reduced HepG2 and Hep3B cell invasion (Figure 2B). Compared with naïve hCEC-Exo, hCEC-Exo-214, in combination with oxaliplatin or sorafenib, had a more robust effect on reduction of HepG2 and Hep3B cell invasion (Figure 2B).

Collectively, these data indicate that hCEC-Exo enhance the anti-HCC effect of oxaliplatin and sorafenib, and that engineered hCEC-Exo-214 have a more potent anti-HCC effect than naïve hCEC-Exo.

### Engineered hCEC-Exo-214 sensitize patient tumor-derived primary cells to anti-cancer drugs

**Figure 3:**
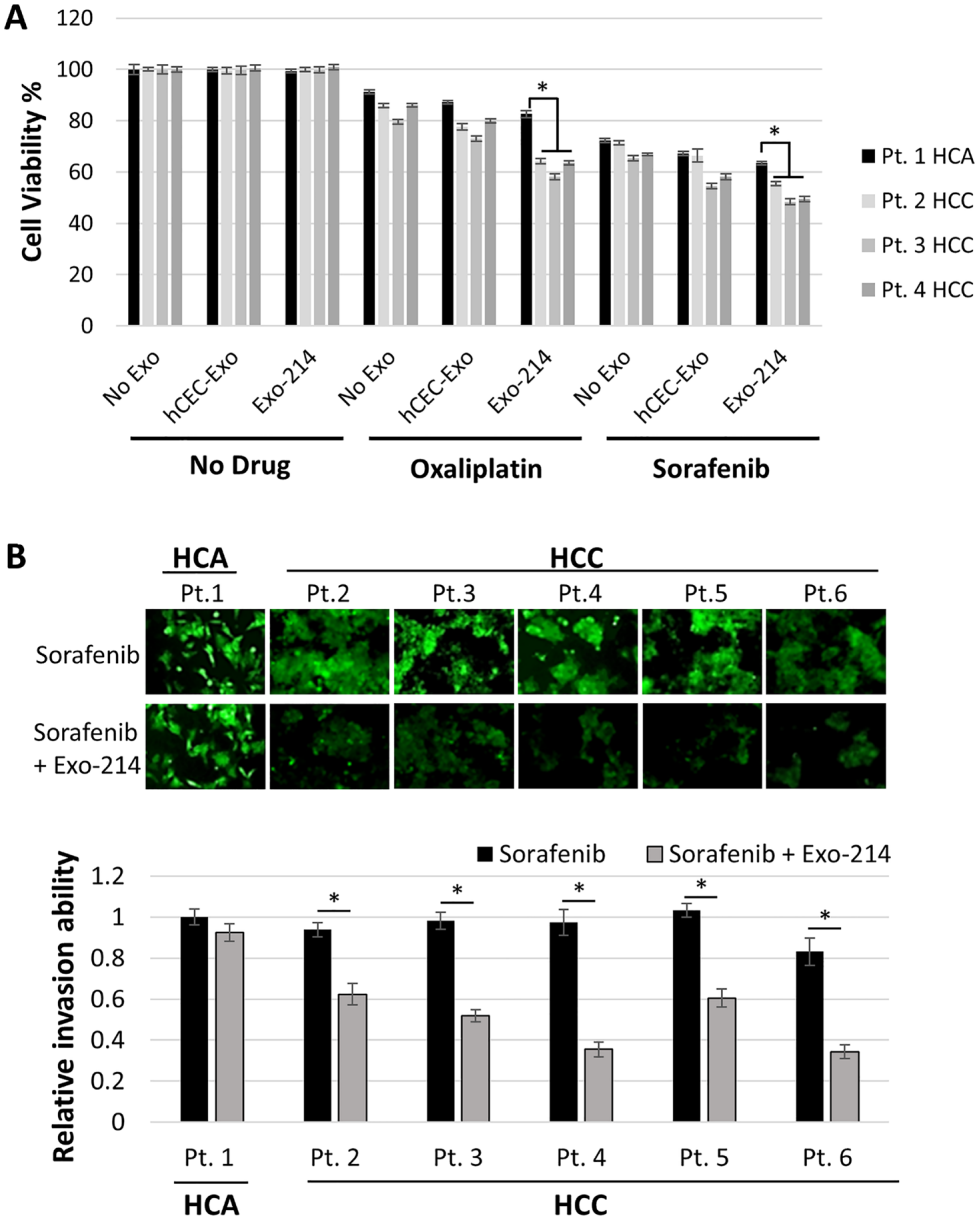
The effect of the combination therapy on tumor primary cells derived from patients with liver tumor. (**A**) Quantitative data from MTT assay showed the effect of hCEC-Exo or hCEC-Exo-214 in combination with oxaliplatin or sorafenib on cell viability of primary cells derived from tumor tissues of four individual patients (Pt) with hepatocellular adenoma (HCA) or hepatocellular adenoma (HCC). Data are presented as mean ± SD. *N* = 5. (**B**) Representative microscopic images and quantitative invasion data showed the effect of hCEC-Exo or hCEC-Exo-214 in combination with sorafenib on cell invasion of primary cells derived from tumor tissues of six individual patients with HCA or HCC. Values are expressed in mean ± SEM. *N* = 3. ^*^ *P* < 0.001.

The effect of hCEC-Exo-214, in combination with oxaliplatin or sorafenib, was evaluated in six patient-derived tumor cells. Patient 1 had a hepatocellular adenoma (HCA) which is a benign liver tumor. Patients 2–6 had HCC. Compared to anti-cancer drugs alone, naïve hCEC-Exo or hCEC-Exo-214, in combination with oxaliplatin or sorafenib, significantly reduced the viability of patient-derived tumor cells from all three individual patients with HCC (patients 2–4) tested. hCEC-Exo-214 were significantly more effective than naïve hCEC-Exo in sensitizing HCC to anti-tumor drugs (Figure 3A). Sorafenib is the FDA approved, first-line treatment for advanced hepatocellular carcinoma. We thus, examined the effect of hCEC-Exo-214 in combination with sorafenib on cell invasion of patient tumor-derived primary cells. The combination treatment significantly inhibited primary cell invasion compared with sorafenib treatment alone (Figure 3B). Interestingly, the significant effects of the combination treatment on cell viability and invasion were not detected in primary tumor cells from the patient with HCA (Figure 3A and 3B).

These data suggest that the results of the combination treatment on HepG2 and Hep3B cell lines are more applicable to patient-derived tumor cells from HCC patients than from the HCA patient.

### Priming with hCEC-214-Exo sensitizes HCC cells to the anti-tumor effect of therapeutic drugs

**Figure 4:**
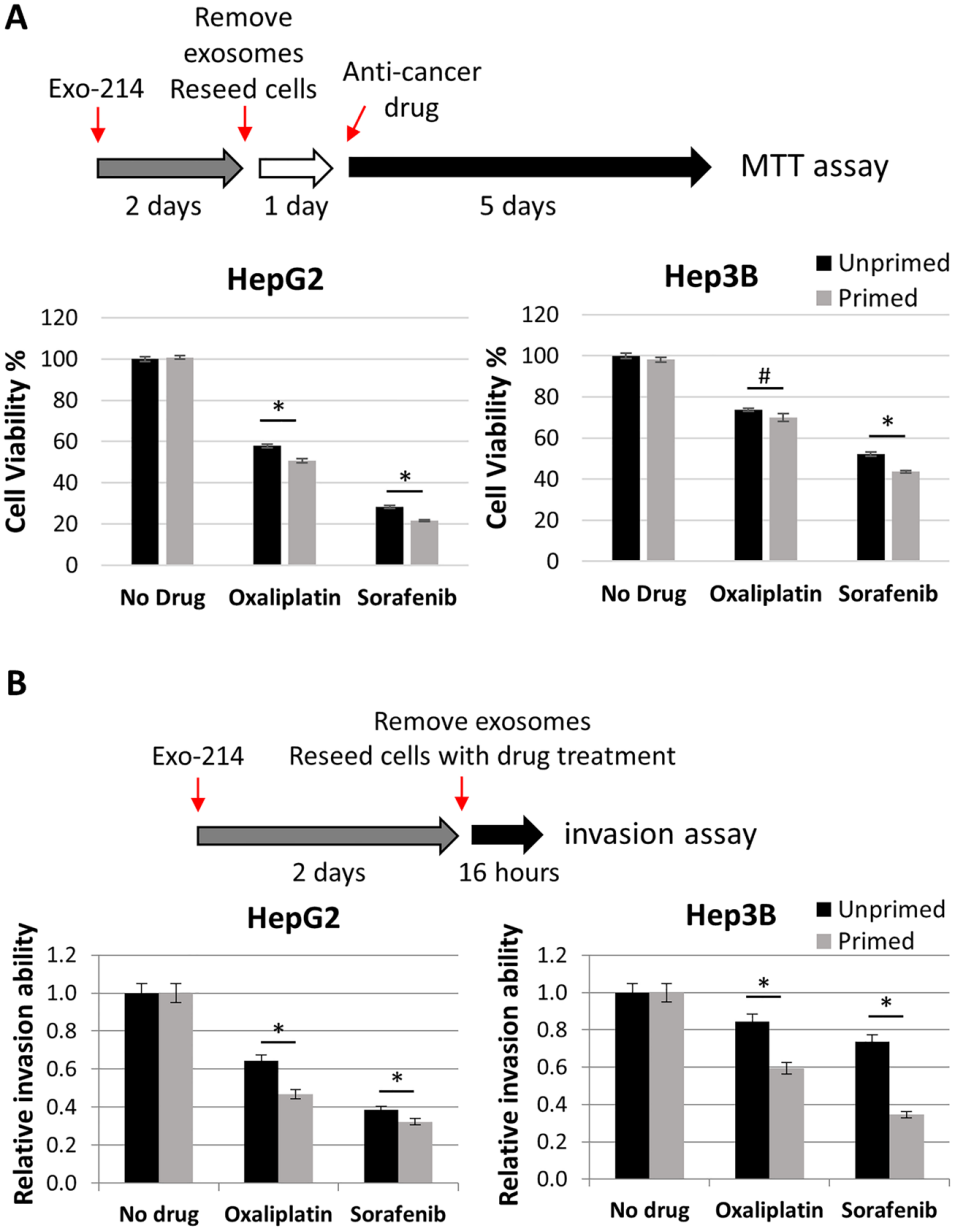
The effect of priming HCC cells with hCEC-Exo-214 on anti-cancer drug treatment. (**A**) Schematic illustration of priming experimental protocol of cell viability and quantitative data of cell viability of primed HepG2 and Hep3B cells. Data are presented as mean ± SD. *N* = 5. (**B**) Schematic illustration of priming experimental protocol of cell invasion and quantitative data of cell invasion of primed HepG2 and Hep3B cells. Values are expressed in mean ± SEM. *N* = 3. ^#^ *P* < 0.05, ^*^ *P* < 0.001.

Tumor cell-derived exosomes are essential to metastatic niche formation, tumor development and progression [52]. Although aforementioned data indicate that the hCEC-Exo-214 alone did not impact HCC cell viability and invasion, we examined whether priming HCC cells with exosomes affects anti-cancer drug efficacy in HCC cells. HepG2 and Hep3B cells were treated with hCEC-Exo-214 for two days, after which culture medium was replaced with fresh medium without hCEC-Exo-214. Oxaliplatin or sorafenib were subsequently applied to the exosome-primed cells. Compared to non-primed HCC cells, oxaliplatin or sorafenib significantly reduced cell viability of HepG2 and Hep3B cells primed with hCEC-Exo-214, although the reduction was marginal (Figure 4A). Considering that cell viability was assayed 6 days after hCEC-Exo-214 priming, the marginal yet significant difference observed (Figure 4A) is of note. However, oxaliplatin or sorafenib more profoundly reduced HepG2 and Hep3B cell invasion in hCEC-Exo-214 primed HCC cells (Figure 4B). Collectively, these priming data suggest that priming HCC cells with hCEC-Exo-214 enhances the anti-cancer drug efficacy of oxaliplatin and sorafenib.

### The combination of hCEC-Exo-214 with anti-cancer therapy reduces protein levels of P-glycoprotein and splicing factor 3B subunit 3 in HCC cells

**Figure 5:**
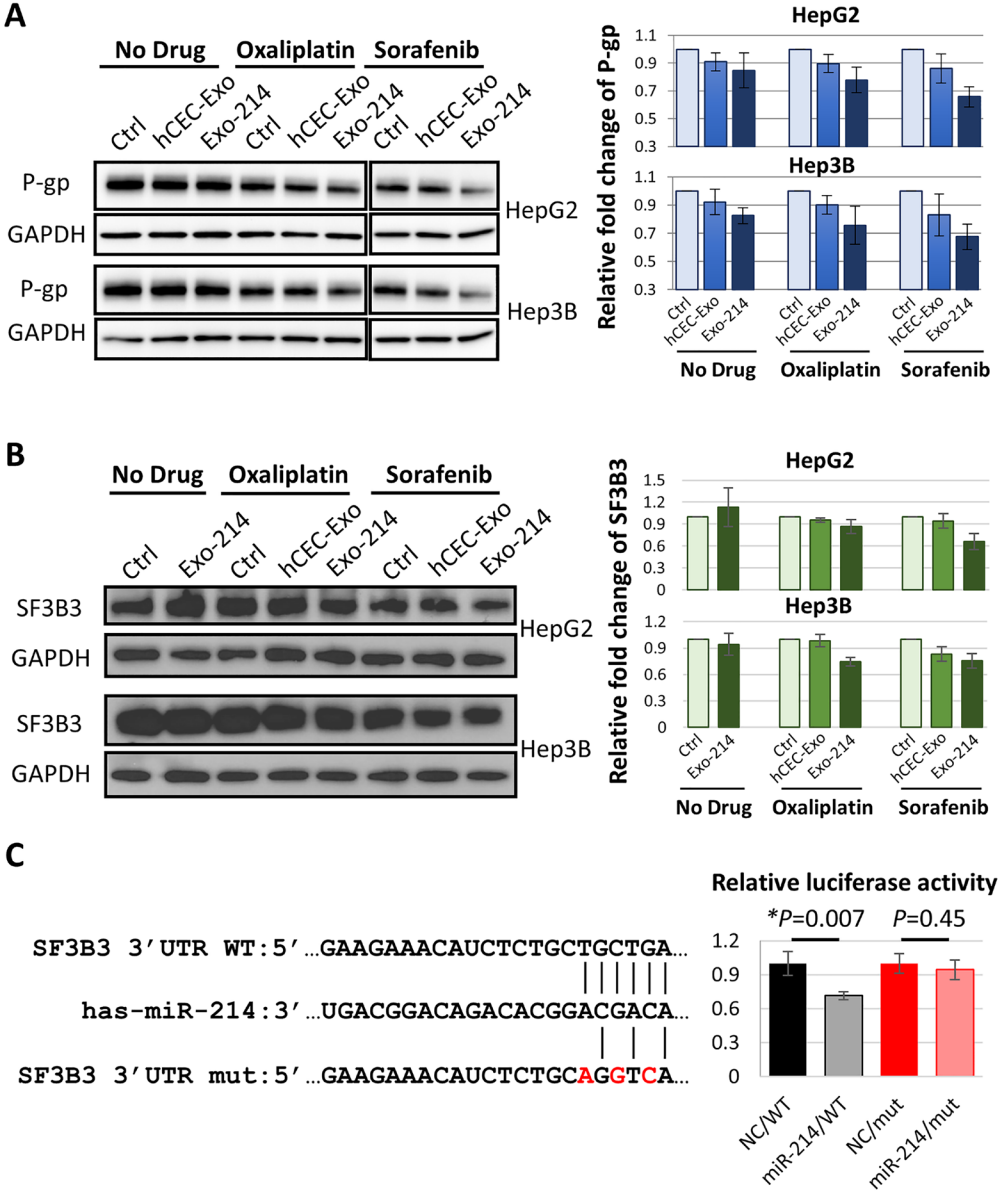
The effect of hCEC-Exo-214 in combination with anti-cancer drugs on P-gp and SF3B3 in HCC cells. (**A** and **B**) Representative Western blot images and quantitative data of protein levels of P-gp (A) and SF3B3 (B) in HepG2 and Hep3B cells 2 days after treatment. Blot images of sorafenib treatment set were combined with no drug and oxaliplatin treatment set. Western blot band intensity was quantified by using ImageJ with normalization to GAPDH. Values are expressed in mean ± SEM. *N* = 3. (**C**) Dual-luciferase activity assay. Putative miR-214-3p target sequence of SF3B3 3′-UTR region were listed. WT: wildtype. mut: mutant. Assay was performed in HepG2 cells. NC: negative control. Values are normalized and are presented in mean ± SEM. *N* = 3 individual experiments.

P-glycoprotein (P-gp), encoded by the adenosine triphosphate (ATP)-binding cassette subfamily B member 1 (ABCB1) gene, is one of the genes known to facilitate HCC chemoresistance [27]. Western blot analysis revealed that, compared to drug alone, hCEC-Exo-214 in combination with oxaliplatin or sorafenib significantly reduced P-gp protein levels in HepG2 and Hep3B cells (Figure 5A). The effect of the combination treatment on reduction of P-gp appears specific because the combination treatment did not affect levels of multidrug resistance-associated protein 2 (MRP2) (Supplementary Figure 3). Additionally, we found that hCEC-Exo-214 in combination with oxaliplatin or sorafenib significantly reduced protein levels of splicing factor 3B subunit 3 (SF3B3) (Figure 5B). SF3B3 is a putative target for miR-214 as predicted by TargetScan (version 7.2, http://www.targetscan.org) (Figure 5C). To verify whether miR-214 targets SF3B3, a dual-luciferase assay was performed. Co-transfection with the wildtype (WT) 3′-UTR region of SF3B3 with miR-214 mimics significantly reduced luciferase activity to ~71.5% of that seen with a negative control (NC). This is comparable to reduced levels of other genes targeted by miR-214 [14, 15, 17]. In addition, miR-214 mimics did not significantly reduce luciferase activity when SF3B3 3′-UTR was mutated (Figure 5C). These data suggest that miR-214 targets SF3B3 3′-UTR. Collectively, these data suggest that reduction of P-gp and SF3B3 proteins may contribute to the observed beneficial effect of combination treatment on HepG2 and Hep3B cells.

## DISCUSSION

The present study shows that naïve hCEC-Exo and engineered hCEC-Exo-214 substantially sensitize HCC cells to the effects of traditional anti-cancer agents. The impact was significantly more potent with hCEC-Exo-214 than with hCEC-Exo. The effect was seen on both HCC cell viability and invasion (Figure 2). The amplified benefit of the combination therapy was observed in multiple HCC cell lines and was also seen in primary cancer cells from multiple patients with HCC. The effect was present with two drugs with divergent mechanisms of action and was specific to neoplastic tissue. These *in vitro* data demonstrate, for the first time, that hCEC-Exo, particularly exosomes carrying elevated miR-214, can enhance the anti-tumor effect of oxaliplatin or sorafenib against HCC.

Tumor-derived extracellular vesicles (EVs), including exosomes, mediate cancer formation, progression, and metastasis by facilitating communication between the cancer niche and distal cells [53]. For example, tumor-derived exosomes induce epithelial-mesenchymal transition (EMT) [54] and chemoresistance [55, 56]. Compared to studies using tumor-derived EVs as biomarkers of cancer presence or progression, investigations of exogenous exosomes as therapeutic agents in cancer are more limited. EVs derived from human liver stem cells suppress growth of HepG2 and primary HCC cells [57], while EVs derived from human bone marrow mesenchymal stem cells inhibit *in vitro* cell cycle progression of several tumor cell lines, including HepG2, and *in vivo* progression of subcutaneous tumors in SCID mice [58].

The present study shows that naïve hCEC-Exo and engineered hCEC-Exo-214 sensitize HCC cells to the anti-cancer effects of sorafenib and oxaliplatin as measured by cell viability and invasion assays (Figures 2 and 3). These findings are further supported by priming data in which HCC cells, pre-treated with naïve hCEC-Exo or engineered hCEC-Exo-214, experience an enhanced anti-tumor effect of oxaliplatin or sorafenib. Monotherapy with naïve hCEC-Exo or engineered hCEC-Exo-214 did not significantly affect HCC cell viability or invasion (Figure 4). The therapeutic effect is specific to HepG2 and Hep3B cells in that the combination therapy did not affect the viability or invasion of the hepato-epithelial cell line, THLE-2 (Supplementary Figure 2). Importantly, the anti-tumor effect of the combination therapy was observed in primary cancer cells derived from patients with HCC but not in tumor cells derived from a patient with HCA (Figure 3). These data suggest that the combination therapy preferentially targets HCC cells, which is consistent with evidence that the interaction of exosome cargo with recipient cells is cell type dependent [59]. Taken together, the present study suggests a potential therapeutic role for exosomes derived from human cerebral endothelial cells to enhance traditional anti-cancer drug treatment in HCC.

The use of exosomes to deliver miRNAs for treatment of HCC has been investigated. Exosomes carrying miR-451a [41], miR-125b [42], miR-122 [43] and miR-26 [40] suppress HCC growth. However, the role of miR-214 in mediating cancer progression has been investigated with inconsistent results. In one study, miR-214 was found to have increased expression in breast cancer tissue which contributed to cell invasion [60, 61]. A conflicting study found decreased levels of miR-214 in breast cancer tissue leading to increased cell proliferation and invasion [60, 61]. In addition, miR-214 negatively regulates liver metastasis of colorectal cancer [18], but contributes to tumor progression and metastasis in melanoma [62]. In HCC, overexpression of miR-214 in HepG2, Hep3B, HLE, and SK-HEP-1 cells suppresses cell growth and invasion [14, 19]. Ectopic expression of miR-214 in SK-HEP-1 cells inhibits xenograft tumor formation and decreases microvascular density in tumor-bearing nude mice [14]. In this study, using qRT-PCR analysis, miR-214 was found to be downregulated in HCC cell lines and in primary patient HCC cells, which validates reports by others [14–16]. Using multiple experimental approaches, the present study, for the first time, demonstrates that exosomes carrying elevated miR-214 enhance anti-cancer drug sensitivity in HCC cells.

Therapeutically engineered exosomes carrying elevated miR-214, hCEC-Exo-214, robustly and specifically amplify anti-cancer drug efficacy in HCC cell lines and in patient-derived HCC cells. We do not rule out that exosomes from other cells may have comparable activity, in this regard, but we chose to evaluate hCEC-Exo because we found, in preliminary studies, they were superior to mesenchymal stem cell exosomes in mitigating chemotherapy induced neurotoxicity and in enhancing antineoplastic drug activity (data not shown). Although miRNA-214 has been shown to target p53 in breast cancer [60], this is not likely the mechanism by which hCEC-Exo-214 exert their effect in HCC. hCEC-Exo-214 significantly increased anti-cancer drug activity in both HepG2 and Hep3B cell lines. However, HepG2 cells are p53 wildtype allele, whereas Hep3B cells are p53 null allele [63]. Instead, this study suggests that hCEC-Exo-214 target P-gp and SF3B3. Western blot analysis showed significant reduction in protein levels of both P-gp and SF3B3, two genes directly or indirectly targeted by miR-214 (Figure 5). Both genes have been reported to be involved in cancer progression. P-gp mediates chemoresistance in HCC [27, 64]. Increased expression of P-gp was observed in HCC cells resistant to 5-fluorouracil and epirubicin, as well as sorafenib [65, 66]. Reducing P-gp using antisense RNA attenuates doxorubicin resistance in HepG2 cells [67], whereas increased P-gp levels substantially reduces sorafenib efficacy in HCC cells [68]. SF3B3 is a pro-oncogene in renal cancer and knockdown of SF3B3 in renal cancer cells significantly inhibits tumor growth in tumor-bearing mice [69]. SF3B3 also regulates enhancer of zeste 2 polycomb repressive complex 2 subunit (EZH2) that promotes HCC progression [70, 71]. EZH2 is one of the genes directly targeted by miR-214 and overexpression of miR-214 reduces EZH2 protein levels [14]. Thus, reduction of P-gp and SF3B3 by hCEC-Exo-214 is likely to amplify the effect of anti-cancer drugs on HCC cells. Clearly a single miRNA targets numerous mRNAs and an individual mRNA can be targeted by several miRNAs [12, 72]. In addition to miR-214, exosomal cargo contains other miRNAs, proteins and lipids and their roles in mediating the therapeutic effects of the combination therapy cannot be excluded and warrant further investigation.

In summary, our data demonstrate that engineered hCEC-Exo-214 significantly enhance the efficacy of oxaliplatin and sorafenib anti-cancer drugs in HCC. The findings support a potential clinical strategy of combining engineered hCEC-derived exosomes with anti-cancer agents to improve medical therapy in HCC.

## MATERIALS AND METHODS

### Patients and tissues

Patient tissues used in the present study were obtained from patients with hepatocellular carcinoma or hepatocellular adenoma who underwent liver resection or liver transplant at Henry Ford Hospital (Detroit, MI, USA). The study was approved by the Henry Ford Health System (HFHS) Institutional Review Board (IRB No.12996). The IRB grants a waiver of the requirements to obtain informed consent. All experiments were performed in accordance with relevant guidelines and regulations.

### Anti-cancer drugs

Oxaliplatin was purchased from Sigma & Aldrich (St. Louis, MO, USA) and was dissolved in dimethyl sulfoxide (DMSO) to make stock solution at 25 mM. Sorafenib was purchased from Santa Cruz Biotechnology (Dallas, TX, USA), and was dissolved in dimethyl sulfoxide (DMSO) to make stock solution at 100 mM. The stock solutions were further diluted to appropriate concentrations with cell culture medium immediately prior to experiments. DMSO was used as a control.

### Cell culture

Hepatocellular carcinoma cell lines HepG2 (ATCC^®^ HB-8065™), Hep3B (ATCC^®^ HB-8064™), and the human liver epithelial cell line THLE-2 (ATCC^®^ CRL-2706™) were purchased from American Type Culture Collection (ATCC, Rockville, MD) and were cultured in Eagle’s Minimum Essential Medium (EMEM; ATCC). All cell lines were maintained in EMEM containing 10% fetal bovine serum (FBS), 100 units/ml penicillin, 50 μg/ml streptomycin, and 100 μg/ml amphotericin (Invitrogen, Waltham, MA, USA). Cell cultures were maintained in 75 cm^2^ flasks and kept in a humidified atmosphere with 5% CO_2_ at 37°C.

Primary human cerebral endothelial cells (hCEC) (Primary Human Brain Microvascular Endothelial Cells ACBRI 376) were purchased from Cell Systems. The cell culture was maintained using Cell System complete classic medium (4Z0-500) with CultureBoost according to the manufacturer’s protocol.

Liver tumor and adjacent non-tumor tissues were acquired from patients with HCC and liver tumor undergoing liver resection or transplant and the collected tissues were kept in Eagle’s Minimum Essential Medium (EMEM) at 4°C. Under aseptic conditions, the connective tissues, bile ducts, and blood vessels were trimmed away as much as possible. Tumor tissues were then minced into approximate 1 mm pieces and incubated in 6-well plates coated with attachment factor (Cell Systems: 4Z0-210) in EMEM medium 10% fetal bovine serum (FBS) and 1% of anti-anti (Invitrogen). Plates were kept in a humidified atmosphere with 5% CO_2_ at 37°C for 2–3 days to allow attachment. Media was changed regularly, and cells were passed at 80–90% confluency. All experiments were done within 5–10 cell passages.

### Transfection of miR-214 into hCEC cells

The human pre-microRNA expression construct Lenti-miR-214 (PMIRH214PA-1) was purchased from System Biosciences. Briefly, 5 × 10^5^ hCECs were suspended in 100 μl of Ingenio Electroporation Solution (Mirus Bio LLC., Madison, WI, USA) with 3 μg of plasmid DNA. Program U11 was used for electroporation in an Amaxa Nucleofector Device (Lonza Group Ltd., Walkersville, MD, USA). Transfected hCECs were re-suspended in 10 ml complete culture medium, followed by centrifugation at 500× g, and then cultured for exosome production. 1 × 10^6^ hCECs were seeded in 10 ml complete classic medium (4Z0-500, Cell Systems, Kirkland, WA, USA) and cultured for 48 hr. The culture medium was then replaced with serum free medium (4Z3-500, Cell Systems, Kirkland, WA, USA), and cells were cultured for an additional 48 hr when engineered exosomes were collected for analysis and experiments.

### Isolation and characterization of exosomes from naïve and engineered hCECs

Exosomes derived from hCECs or miR-214 transfected hCECs were isolated from the supernatant of CECs cultured in exosome free medium according to our published protocol [73]. Briefly, the culture medium of hCECs was collected after a 48~72 h incubation period according to time required to reach 80% confluence, followed by centrifugation at 2,000 rpm for 5 min. The supernatant of the spin down was filtered through 0.22 μm filter (Millipore, CA, USA) to remove dead and large cell debris. The flow through was then centrifuged at 100,000X*g* for 2 hours. The pellet was dissolved in 200 μl sterilized PBS, and the supernatant was collected as a negative control. The particle numbers and size of harvested hCEC-exosomes were analyzed by Nanoparticle Tracking Analysis (NTA) system (IZON, UK). Transmission electron microscope (TEM) and Western blot analysis were performed to characterize hCEC-exosomes [74]. Total proteins from hCEC-exosomes were collected using 2X lysis buffer (RIPA, Sigma) supplemented with Protease Inhibitor Cocktails set I (100X) (MilliporeSigma, Burlington, MA, USA) followed by Western Blot analysis of exosomal marker proteins.

### MTT (3-(4,5-dimethylthiazol-2-yl)-2,5 diplenyltetrazolium bromide) assay

To measure cell viability, cells were seeded in 96-well plates at a density of 800 cells per well. After overnight incubation, culture medium was removed, and cells were rinsed with PBS and incubated in complete medium with indicated treatment. After 5 days of treatment, medium was removed and MTT was added to each well with an additional 4 hr incubation to allow mitochondrial dehydrogenase to convert MTT into insoluble formazan crystals. The medium was then aspirated, and formazan solubilized by adding 150 μl of DMSO. The absorption of solubilized formazan was measured at a wavelength of 490 nm by an ELISA plate reader (EL340 microplate reader; Bio-Tek Instruments, Winooske, VT, USA).

### Invasion assay

Invasion assay was performed according to our published protocol with modification [75]. Specifically, 24-well Plate invasion chambers were pre-coated with Matrigel (Corning, Corning, NY, USA). HepG2 or Hep3B cells (5 × 10^4^), after receiving indicated treatment for 2 days, were re-suspended in 0.5 ml of serum-free medium and loaded to the upper chamber while the lower chamber was filled with 0.5 ml of complete medium containing FBS, which served as a chemo-attractant. After 16 hr of incubation at 37°C, cell ability to penetrate the extracellular matrix (ECM) was assessed by staining the cells on the lower surface of the membrane with CellTracker™ Green (Molecular Probes, Eugene, OR, USA). Four fields of cells were counted randomly in each well under a fluorescent microscope at 200× magnification. Data are normalized to control treatment, which is set as 1, and are expressed as mean ± SEM of three independent experiments.

### Western blot analysis

HepG2 and Hep3B cells (5 × 10^5^ cells) were seeded in T25 flasks. One day after seeding, cells were subjected to indicated treatment for 48 hr. Cells were rinsed with ice cold PBS followed by extraction in 500 μl RIPA lysis buffer (Life Technologies, Carlsbad, CA, USA). Equal amounts of proteins, as determined by the BCA protocol (Pierce, Rockford, IL, USA), were run on 10% Tris-Glycine gels (Invitrogen) and then transferred to PVDF membranes (Whatman). The membranes were blocked with 0.1% I-Block (Applied Biosystems, Foster, CA, USA) in PBS-T (0.1% Tween-20), followed by incubation with primary antibodies against P-gp (ab170904, Abcam), MRP2 (R260, Cell Signaling), SF3B3 (ab209402, Abcam), GAPDH (ab9484, Abcam), CD9 (ab92726, Abcam), CD63 (ab134045, Abcam), and Alix (3A9, Cell Signaling). Bands were detected using SuperSignal West Pico chemiluminescent protein detection kits (Pierce). Each experiment was repeated three times. The densities of the bands were analyzed using ImageJ software.

### Isolation of total RNA and quantitative reverse transcribed-PCR

After 2 days of treatment, HepG2 and Hep3B cells were lysed in Qiazol reagent, and the total RNA was isolated using miRNeasy Mini kits (Qiagen, Valencia, CA, USA). miRNA was stem-loop reverse transcribed (RT) with the miRNA Reverse Transcription kit (Applied Biosystems) and real-time PCR amplification was performed with the TaqMan miRNA assay (Cat# 4427975, Applied Biosystems), which is specific for mature miRNA sequences. U6 snRNA was used as the internal control for TaqMan miRNA assay to detect the expression level changes of miR-214 in cells. Primers used for miR-214-3p (Assay ID: 002306), miR-92-3p (Assay ID: 000431), U6 (Assay ID: 001973).

### Dual-luciferase activity assay

Luciferase reporter assay was conducted according to previous report [76]. Briefly, HepG2 cells were cultured in 6-well plates and co-transfected with 3 μmol of pMIR-REPORT (Applied Biosystems) containing either SF3B3-3′UTR-WT or SF3B3-3′UTR-mut, 30 pmol of miR-214-3p mimics (Cat#:4464066. ThermoFisher Scientific, Waltham, MA) or negative control (Cat#:4464058), and 1μmol of pRL-TK (Promega, Madison, WI, USA) containing Renilla luciferase per well. Transfection was performed by means of Lipofectamine 2000 (Invitrogen) and Opti-MEM I reduced serum medium (Invitrogen). Three days after transfection, firefly and Renilla luciferase activity was analyzed by using the Dual-Luciferase Reporter Assay kit (Promega) and plate reader (Perkin Elmer, Waltham, MA, USA). Data were presented as relative luciferase activity. Three independent experiments were performed.

### Statistical analysis

Data are presented as mean and standard error. Statistical significance was analyzed by Student *T*-test. *P* value less than 0.05 (*P* < 0.05) was considered significant.

## SUPPLEMENTARY MATERIALS



## Abbreviations

HCC: hepatocellular carcinoma
HCA: hepatocellular adenoma
hCEC: human cerebral endothelial cells
Exos: exosomes
Sup: supernatant
hCEC-Exo: hCEC-derived exosomes
hCEC-Exo-214: engineered hCEC-derived exosomes carrying elevated miR-214
P-gp: P-glycoprotein
SF3B3: splicing factor 3B subunit 3
Pt.: patient
