# Supplementary material for: MicroRNA-214 enriched exosomes from human cerebral endothelial cells (hCEC) sensitize hepatocellular carcinoma to anti-cancer drugs
Source: Oncotarget. 2021 Feb 2;12(3):185–98. doi: 10.18632/oncotarget.27879 (PMC7869574; doi:10.18632/oncotarget.27879)
Supplement: Supplementary file 1 [file oncotarget-12-185-s001.pdf]

MicroRNA-214 enriched exosomes from human cerebral endothelial cells (hCEC) sensitize hepatocellular carcinoma to anti-cancer drugs

SUPPLEMENTARY MATERIALS

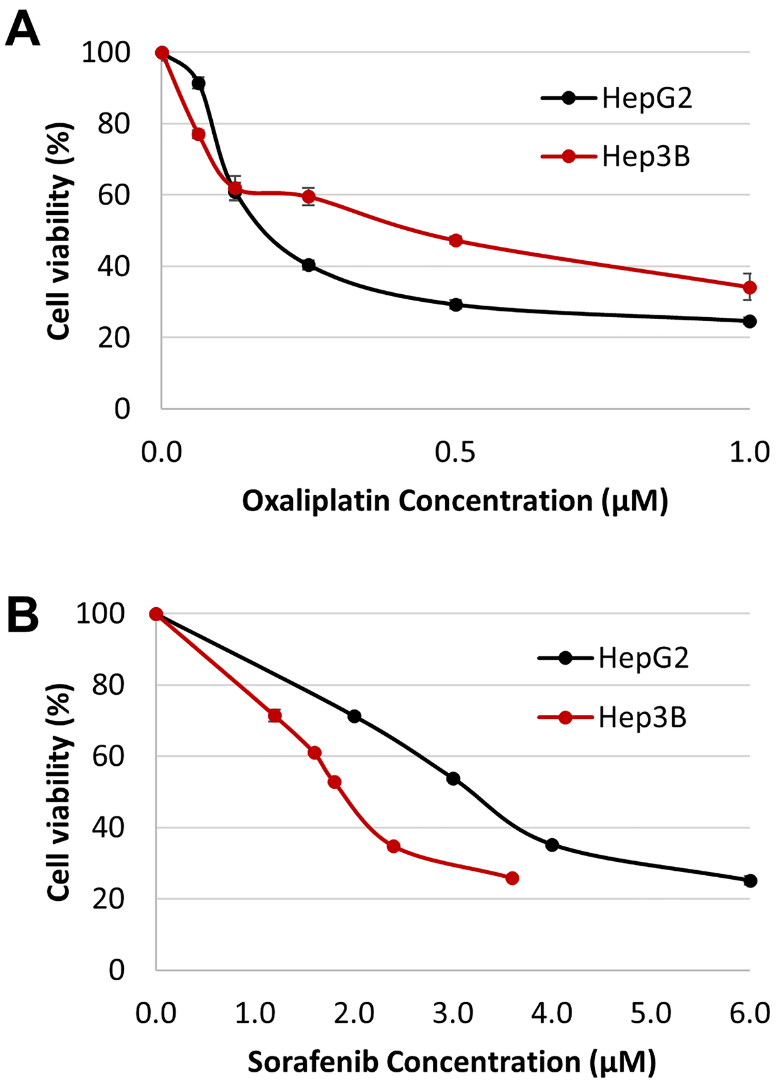

Supplementary Figure 1: Cell viability of HepG2 and Hep3B cells treated with different concentrations of anti-cancer drugs. MTT assay analysis showed cell viability of HepG2 and Hep3B cells treated with oxaliplatin (**A**) and sorafenib (**B**). Data are presented as mean ± SD. *N* = 5.

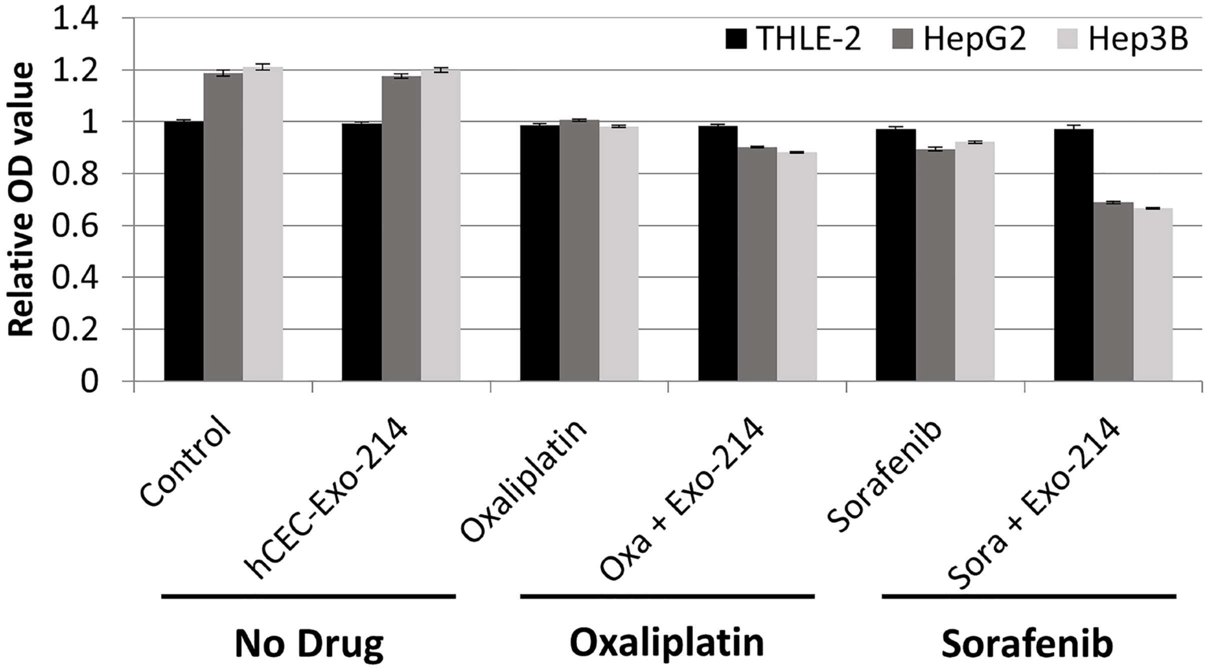

Supplementary Figure 2: THLE-2 is insensitive to either drug treatments, or the combinatorial treatment of anti-cancer drug + hCEC-Exo-214. Cell viability of THLE-2, HepG2, and Hep3B cells are determined 2 days after treatment by MTT assay. $N = 5$. Exo-214 at 10^8 particles/ml were applied alone or in combination with either oxaliplatin (0.0625 µM) or sorafenib (0.8 µM). All OD values are normalized to THLE-2 control value. Data are presented as mean ± SD. $N = 5$.

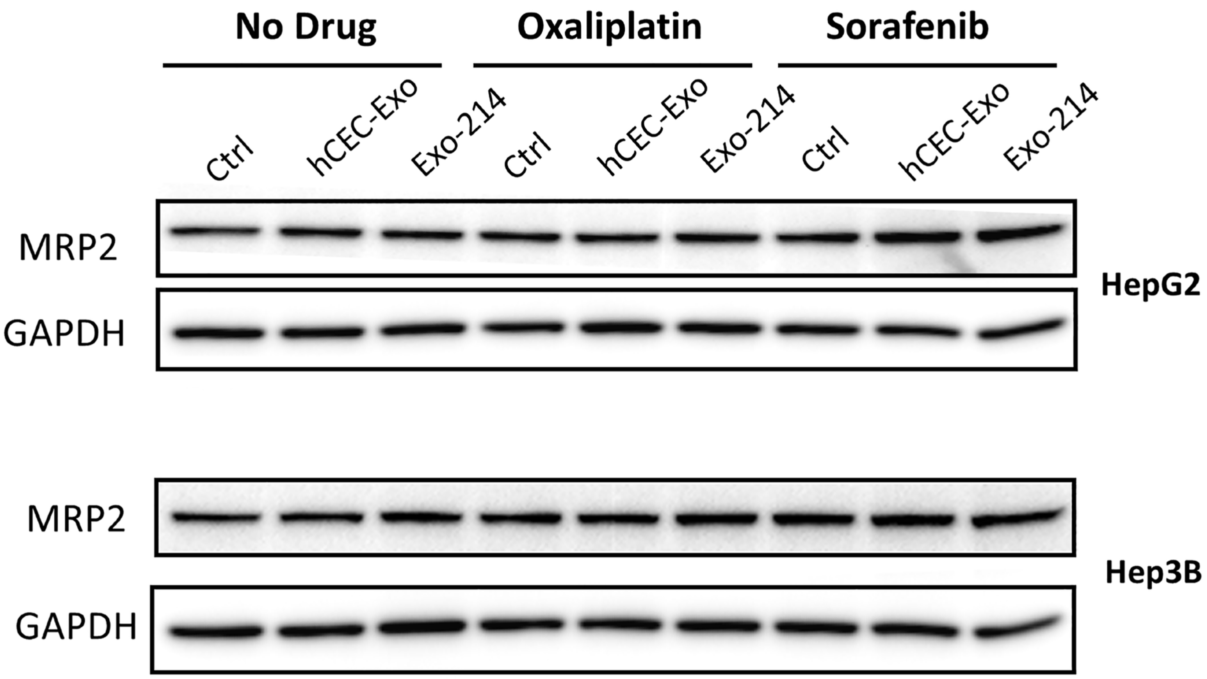

Supplementary Figure 3: The effect of hCEC-Exos or hCEC-Exo-214 in combination with anti-cancer drugs on MRP2 in HCC cells. Representative Western blot images showed protein levels of MRP2 in HepG2 and Hep3B cells 2 days after treatment.
